# How to start medication for ADHD?

**DOI:** 10.1192/j.eurpsy.2025.223

**Published:** 2025-08-26

**Authors:** O. Kilic

**Affiliations:** Department of Neurosciences, Bezmialem Vakif University, Institute of Health Sciences, Istanbul, Türkiye

## Abstract

**Abstract:**

Starting medication for attention deficit and hyperactivity disorder (ADHD) in adults begins with a thorough evaluation. Diagnostic evaluation should involve a careful investigation of the psychiatric and medical comorbidities and an interview with the patient and family members or partners. The next step is to assess the severity of ADHD symptoms, comorbidities and their impact on functioning. This will help guide the order in which targeted treatments are introduced. When selecting the medication, considerations such as the presentation of ADHD symptoms, the patient’s medical history, previous responses to ADHD treatments, and preferences about the effect onset are crucial. This choice typically lies between stimulant and non-stimulant options. The initial dose prescribed should be low enough to mitigate potential side effects while still effectively alleviating symptoms and gradually improving functioning. Treating comorbid psychiatric disorders, such as depression, anxiety, and substance use disorders, is equally important alongside ADHD treatment. The dosage should be slowly adjusted based on the patient’s response and any side effects that arise. Regular follow-up appointments are essential during this phase to monitor the medication’s effectiveness, identify side effects, and make necessary dosage adjustments. Dr Ozge Kilic will discuss key tips related to assessment, medication selection, titration, adjusting dosages, and managing side effects through clinical case vignettes.

**Disclosure of Interest:**

None Declared

